# Standardised criteria for classifying the International Classification of Activities for Time-use Statistics (ICATUS) activity groups into sleep, sedentary behaviour, and physical activity

**DOI:** 10.1186/s12966-019-0875-5

**Published:** 2019-11-14

**Authors:** Nucharapon Liangruenrom, Melinda Craike, Dorothea Dumuid, Stuart J. H. Biddle, Catrine Tudor-Locke, Barbara Ainsworth, Chutima Jalayondeja, Theun Pieter van Tienoven, Ugo Lachapelle, Djiwo Weenas, David Berrigan, Timothy Olds, Zeljko Pedisic

**Affiliations:** 1 Institute for Health and Sport, Victoria University, Melbourne, Australia; 2 Institute for Population and Social Research, Mahidol University, Nakhon Pathom, Thailand; 3 Mitchell Institute, Victoria University, Melbourne, Australia; 4 Alliance for Research in Exercise, Nutrition and Activity, School of Health Sciences, University of South Australia, Adelaide, Australia; 5 Institute for Resilient Regions, University of Southern Queensland, Springfield, Australia; 6 College of Health and Human Services, University of North Carolina at Charlotte, NC, USA; 7 Department of Kinesiology, Shanghai University of Sport, Shanghai Shanghai, People’s Republic of China; 8 College of Health Solutions, Arizona State University, Phoenix, AZ USA; 9 Faculty of Physical Therapy, Mahidol University, Nakhon Pathom, Thailand; 10 Research Group TOR, Department of Sociology, Vrije Universiteit Brussel, Brussels, Belgium; 11 Social Policy Research Centre, University of New South Wales, Sydney, Australia; 12 Department of Urban Studies and Tourism, Universite du Quebec a Montreal, Montreal, Canada; 13 Research Group Interface Demography, Department of Sociology, Vrije Universiteit Brussel, Brussels, Belgium; 14 Behavioral Research Program, Division of Cancer Control and Population Sciences, National Cancer Institute, Bethesda, MD USA

**Keywords:** ICATUS, Time-use survey, Physical activity, Sedentary behaviour, Sleep, Time-use epidemiology

## Abstract

**Background:**

Globally, the International Classification of Activities for Time-Use Statistics (ICATUS) is one of the most widely used time-use classifications to identify time spent in various activities. Comprehensive 24-h activities that can be extracted from ICATUS provide possible implications for the use of time-use data in relation to activity-health associations; however, these activities are not classified in a way that makes such analysis feasible. This study, therefore, aimed to develop criteria for classifying ICATUS activities into sleep, sedentary behaviour (SB), light physical activity (LPA), and moderate-to-vigorous physical activity (MVPA), based on expert assessment.

**Method:**

We classified activities from the Trial ICATUS 2005 and final ICATUS 2016. One author assigned METs and codes for wakefulness status and posture, to all subclass activities in the Trial ICATUS 2005. Once coded, one author matched the most detailed level of activities from the ICATUS 2016 with the corresponding activities in the Trial ICATUS 2005, where applicable. The assessment and harmonisation of each ICATUS activity were reviewed independently and anonymously by four experts, as part of a Delphi process. Given a large number of ICATUS activities, four separate Delphi panels were formed for this purpose. A series of Delphi survey rounds were repeated until a consensus among all experts was reached.

**Results:**

Consensus about harmonisation and classification of ICATUS activities was reached by the third round of the Delphi survey in all four panels. A total of 542 activities were classified into sleep, SB, LPA, and MVPA categories. Of these, 390 activities were from the Trial ICATUS 2005 and 152 activities were from the final ICATUS 2016. The majority of ICATUS 2016 activities were harmonised into the ICATUS activity groups (*n* = 143).

**Conclusions:**

Based on expert consensus, we developed a classification system that enables ICATUS-based time-use data to be classified into sleep, SB, LPA, and MVPA categories. Adoption and consistent use of this classification system will facilitate standardisation of time-use data processing for the purpose of sleep, SB and physical activity research, and improve between-study comparability. Future studies should test the applicability of the classification system by applying it to empirical data.

## Background

Sleep, sedentary behaviour (SB), light physical activity (LPA) and moderate-to-vigorous physical activity (MVPA) are activity-based behaviours associated with a range of health outcomes [1]. For example, short duration of sleep is associated with a higher risk of developing coronary heart disease, stroke, type II diabetes, and certain types of cancer [2–4]. It is suggested that too much SB increases the risk of cardiovascular disease, type II diabetes, and metabolic syndrome [5]. Physical inactivity (usually defined as insufficient amount of MVPA to meet physical activity (PA) recommendations [6]) is also associated with an increased burden of disease, including coronary heart disease, type II diabetes, breast cancer, and colon cancer [7]. Although previous studies examined sleep, SB, LPA, and MVPA as independent predictors of health outcomes, recently, methodological papers suggest these are all mutually exclusive and exhaustive components of the time-finite 24-h day, and should, therefore, be considered as co-dependent variables [8–10]. Recent studies aimed to acknowledge co-dependence of these variables using different analytical approaches, such as isotemporal substitution and compositional data analysis [1, 8, 11–17]. Despite the differences in statistical approaches, there is wide agreement that conceptualising and studying sleep, SB, and PA as integral parts of the 24-h day may lead to novel and important insights into activity-based behaviours and health [8, 10, 18–21]. This new way of conceptualising activity-based behaviours is sometimes referred to as the “Time-Use Epidemiology” paradigm [10].

National time-use surveys have been conducted in over 85 countries worldwide [22]. Time-use survey data have been of great interest for researchers, due to their comprehensiveness and a broad range of possible applications in public health, sociology, economics, and transportation research [23]. It is widely accepted that the validity and reliability of time-use survey data are adequate for large-scale, observational studies [23–29]. Several studies used time-use data to investigate population-level PA patterns [30–36].

Most previous studies in time-use epidemiology have relied on accelerometer-based estimates of sleep, SB, and PA [15, 37–45]. While accelerometers have undoubtedly been providing useful data for time-use epidemiology, they have limitations in terms of validity, generalisability, between-study comparability, and comprehensiveness of movement behaviour estimates [46]. The affordability and sustainability of their use in population surveillance has also been questioned [46]. With complete 24-h data, time-use surveys may be a good alternative to accelerometers, as they also allow researchers to investigate the combined effects of all movement-related behaviours on health [47]. They can also be used to track the prevalence of meeting the new integrative 24-h movement guidelines that include joint recommendations for sleep, SB, and PA [19, 20, 48–51]. However, as time-use surveys were not designed specifically to collect data on PA and SB, their use in time-use epidemiology has been limited. The 24-h movement behaviour data from time-use surveys are, therefore, yet to be explored in detail in relation to health outcomes. To enable this, classification systems for deriving health-related time-use compositions from time-use surveys must be developed and evaluated [25, 52–54]. A recently developed framework entitled Viable Integrative Research in Time-Use Epidemiology (VIRTUE) recognised this as a methodological task of fundamental importance for the further development of time-use epidemiology [10]. The availability of such classification systems is a prerequisite for utilisation of time-use survey data in epidemiological studies on movement-related behaviours.

Response options in time-use surveys are often derived from standardised time-use classification systems. The International Classification of Activities for Time-Use Statistics (ICATUS) is one of the most widely used time-use classification systems. It was developed by the United Nations Statistics Division (UNSD) to provide meaningful and comparable time-use statistics across countries and over time [22, 55, 56]. ICATUS has been used as a framework for several nationally representative time-use surveys, mostly in Asia and Africa [56]. The ICATUS was first introduced as a draft classification in 1997 by the UNSD. In 2000, the expert group carried out further refinements to the activity categories, which was published in 2005 as the Trial ICATUS [55]. Several consultation meetings were organised between 2012 and 2016 among experts and relevant stakeholders to finalise the classification [55]. The ICATUS 2016 is the final version, with a simplified structure and terminologically aligned with existing international standards, such as the System of National Accounts and the International Standard Industrial Classification of All Economic Activities [55]. The Trial ICATUS 2005, a five-level hierarchical classification, is comprised of 15 major divisions, 54 divisions, 92 groups, 200 classes and 363 subclasses. The ICATUS 2016, a three-level hierarchical classification, includes 9 major divisions, 56 divisions, and 165 groups. The Trial ICATUS 2005 has been used in many national time-use surveys since 2000, while the ICATUS 2016 is a finalised classification system for future ICATUS-based time-use surveys [55].

Activity categories from several time-use surveys have previously been classified according to their “Metabolic Equivalent of Task” (MET) [25, 29, 57–61]. One MET describes the human energy expenditure while at rest (i.e., resting metabolic rate or approximately 1 kcal/kg/hour), whilst two METs is twice that at rest [62]. Tudor-Locke and colleagues (2009) assigned MET values to 438 activities in the American Time Use Survey (ATUS) according to the 2011 Adult Compendium of Physical Activities (hereafter called “the Compendium”) [25–27, 63]. Several studies have also applied METs using the Compendium in other time-use surveys, such as the Australian Time Use Survey, Statistics Canada’s General Social Survey – Time Use (GSS-TU), and Belgian Time Use Survey (using Harmonised European Time Use Survey [HETUS] classification) [29, 58–61, 64]. However, no previous studies have developed criteria for classifying ICATUS activities into sleep, SB, LPA, and MVPA categories.

Like other systems that can classify time-use components into different types of health-related domains (e.g. social activities, cognitive activities), a classification system for classifying the ICATUS activities into major activity-based time-use components (i.e., sleep, SB, LPA, and MVPA) would also enable time-use epidemiologists to process data from many existing and future population-representative surveys. Such a system would also facilitate standardisation of data processing in this area, which may improve between-study comparability. To be able to classify time-use components into sleep, SB, LPA and MVPA, one must know: (i) their MET value; (ii) whether they are done while awake or while asleep; and (iii) in which posture they are performed [6, 10]. However, these three criteria have never been inclusively assigned to any time-use surveys. This study, therefore, aimed to assign MET values and codes for wakefulness status and posture to the Trial ICATUS 2005 and the Final ICATUS 2016 activities to enable their classification into sleep, SB, LPA, and MVPA categories. It can be expected that future studies will predominantly use the Final ICATUS 2016. Nevertheless, it should not be disregarded that the Trial ICATUS 2005 has already been used in many national time-use surveys for over a decade, which means a lot of valuable time-use data is already available. To facilitate comparability between studies based on the Trial ICATUS 2005 and the Final ICATUS 2016 and enable research on trends in movement-related behaviours (which are lacking for many countries), we decided to classify activities from both versions.

## Methods

### Classification criteria

Criteria used to classify time into sleep, SB, LPA, and MVPA were: 1) relative energy expenditure (MET values from the Compendium [63]); 2) wakefulness (yes or no); and 3) sitting/reclining/lying posture (yes or no). The answer “no” to sitting/reclining/lying posture implied standing or being on one’s feet while performing an activity. The ICATUS activities were classified into sleep, SB, LPA, and MVPA categories based on the criteria presented in Table 1. Given that a number of ICATUS activity categories are very broad and non-specific, in many cases it would not be possible to make a clear distinction between moderate and vigorous intensity. We, therefore, combined these two intensity levels into MVPA.

**Table 1:** Criteria for classifying time-use components into sleep, SB, LPA, and MVPA

Activity-based category	METs	Wakefulness	Sitting/reclining/lying
Sleep	< 1	No	Yes or No
SB	≥1 – ≤1.5	Yes	Yes
LPA	> 1.5 – < 3	Yes	Yes or No
MVPA	≥3	Yes	Yes or No

Notes: MET: metabolic equivalent of task; SB: sedentary behaviour; LPA: light physical activity; MVPA: moderate-to-vigorous physical activity

### Initial assessment of ICATUS activities

The initial assessment of activities was done for the Trial ICATUS 2005, because the Trial ICATUS provides a more detailed classification activities than the Final ICATUS. The Trial ICATUS 2005 groups activities into five levels. The first level, 2-digit code or “major divisions” includes the broadest groups of activities, and the fifth level, 6-digit code or “subclasses” represents the most detailed level of the classification [65]. The major divisions and their associated subclass activities of the Trial ICATUS 2005 were entered into a separate Excel spreadsheet. One author (NL) conducted an initial assessment by assigning i) relative energy expenditure (MET values from the Compendium); ii) wakefulness status (yes or no); and 3) sitting/reclining/lying posture (yes or no) to each 6-digit activity in each major division of the Trial ICATUS 2005. When assigning the codes, NL consulted the Guide to Producing Statistics on Time Use which provided definitions and descriptions of ICATUS activities, including examples and exceptions [65]. To assign a MET value, each ICATUS subclass activity was matched with one or more Compendium activities according to the examples and descriptions provided in the above-mentioned documents. The coding rules presented in Table 2 were used in the assessment.

**Table 2:** Coding rules to assign Compendium METs, wakefulness, and posture to the ICATUS activities

Coding rule 1	Assign the codes and MET values from the Compendium and the codes for wakefulness and posture to each 6-digit activity		
Coding rule 2	Use a median MET estimate of the respective activities or subcategories	2a.	when more than one activity from the Compendium was assigned to a 6-digit activity
		2b.	when assigning METs to a 4-digit and 5-digit activity
		2c.	when an activity is classified as “not further defined” (n.f.d.) or “not elsewhere classified” (n.e.c.)
		2d.	when there is insufficient information in the explanatory notes; usually classified as “other related activities” and ends in “9”
Coding rule 3	Assign the codes for summary wakefulness and posture to a 4-digit and 5-digit activity according to the assessments made for the majority of its 6-digit subclass activities		

Notes: Compendium: 2011 Adult Compendium of Physical Activities [63]; MET: metabolic equivalent of task; ICATUS: International Classification of Activities for Time-Use Statistics; n.f.d.: not further defined; n.e.c.: not elsewhere classified

The MET values and codes for wakefulness status and posture were assigned to the most detailed level of activities (i.e., subclass activities). For the activities that are broadly described and encompass more than one specific activity in the Compendium, a median MET value of respective Compendium activities was calculated. The summary MET values were also computed for the 4-digit and 5-digit activities in ICATUS 2005 as a median MET value assigned to their subclasses. Summary wakefulness and posture categories were assigned to each 4-digit and 5-digit activity according to the respective assessments made for the majority of its subclasses. The summary assessments were also used for an activity classified as “not further defined” (n.f.d.) or “not elsewhere classified” (n.e.c.) or “other related activities” or ends in “9” activities, where information is insufficient. An extract from the table used in the described assessment process is shown in Table 3, while the complete table can be found in Additional file 1.

**Table 3:** An extract from the table used for the assessment of ICATUS 2005 activities

ICATUS 2005				Assessment			Compendium of Physical Activities		
Code			Description	Summary METs	Wakefulness (Yes/No)	Sitting/reclining/lying (Yes/No)	Code	Major heading: specific activities	METs
1211		Visual, literary and performing arts (as hobby) and related courses		2.75 (median of four subclass activities)	yes	no			
	12111	121110	Visual arts	2.75 (median of respective Compendium activities)	yes	yes	09020	Miscellaneous: drawing, writing, painting, standing	1.80
							09075	Miscellaneous: sitting, arts and crafts, carving wood, weaving, spinning wool, light effort	1.80
							09080	Miscellaneous: sitting, arts and crafts, carving wood, weaving, spinning wool, moderate effort	3.00
							09085	Miscellaneous: standing, arts and crafts, sand painting, carving, weaving, light effort	2.50
							09090	Miscellaneous: standing, arts and crafts, sand painting, carving, weaving, moderate effort	3.30
							09095	Miscellaneous: standing, arts and crafts, sand painting, carving, weaving, vigorous effort	3.50
	12112	121120	Literary arts	1.30 (median of respective Compendium activities)	yes	yes	09040	Miscellaneous: sitting, writing, desk work, typing	1.30
							09060	Miscellaneous: sitting, studying, general, including reading and/or writing, light effort	1.30
							07050	Inactivity quiet/light: reclining, writing	1.30
	12113	121130	Performing arts (dance, music, theatre)	4.00 (median of respective Compendium activities)	yes	no	03031	Dancing: general dancing (e.g. disco, folk, Irish step dancing, line dancing, polka, contra, country)	7.80
							03010	Dancing: ballet, modern, or jazz, general, rehearsal or class	5.00
							10074	Music playing: playing musical instruments, general	2.00
							10130	Music playing: marching band, baton twirling, walking, moderate pace, general	4.00
							10131	Music playing: marching band, playing an instrument, walking, brisk pace, general	5.50
							10135	Music playing: marching band, drum major, walking	3.50
							11870	Occupation: working in scene shop, theater actor, backstage employee	3.00
	1211x		Visual, literary and performing arts n.f.d.	2.75 (summary assessments)	yes	no			

Notes: Compendium: 2011 Adult Compendium of Physical Activities [63]; MET: metabolic equivalent of task; ICATUS 2005: Trial International Classification of Activities for Time-Use Statistics 2005; n.f.d.: not further defined

MET values and the codes for wakefulness and posture were not assigned to occupational and travel-related activities, because insufficient information is provided in the Guide to Producing Statistics on Time Use [65] and the ICATUS 2016 document [55] to be able to make an informed assessment of these ICATUS activities.

### Harmonisation of ICATUS 2005 and 2016 activities

Once all subclass activities of the Trial ICATUS 2005 were coded, one author (NL) matched 3-digit activities (the most detailed level) from the ICATUS 2016 with corresponding activities of the Trial ICATUS 2005, where applicable. The description of the activity codes in the Trial ICATUS 2005 and the ICATUS 2016 [55, 65], including examples and exceptions, was examined for harmonisation purposes. The MET values, wakefulness status, and posture categories assigned to ICATUS 2005 activities were used for their matching ICATUS 2016 activities. For the ICATUS 2016 activities that could not be matched with any ICATUS 2005 activity, we assigned MET values, wakefulness status, and posture separately. Furthermore, some ICATUS 2016 activities were matched with multiple ICATUS 2005 activities. To such activities we also assigned MET values, wakefulness status, and posture separately. An extract from the table used in the described harmonisation process is shown in Table 4, while the complete table can be found in Additional file 1.

**Table 4:** An extract from the table used for the harmonisation of ICATUS 2005 and 2016 activities

ICATUS 2005				ICATUS 2016	
Code			Description	Code	Description
1511			Sleep and related activities		
	15111	151110	Night sleep/essential sleep	911	Night sleep/essential sleep
	15112	151120	Incidental sleep/naps	912	Incidental sleep/naps
	15113	151130	Sleeplessness	913	Sleeplessness
	1511x		Sleep and related activities n.f.d.	919	Other sleep and related activities
		03111	Processing of food products	127	Making and processing goods for the market in household enterprises
		03112	Making of other food products and beverages		
		03113	Making textiles, wearing apparel, leather and associated products		
		03114	Craft-making using all types of materials		
		03115	Tobacco preparing and curing		
		03116	Making bricks, concrete slabs, hollow blocks, tiles etc.		
		03117	Making herbal and medicinal preparations		

Notes: ICATUS 2005: Trial International Classification of Activities for Time-Use Statistics 2005; ICATUS 2016: International Classification of Activities for Time-Use Statistics 2016; n.f.d.: not further defined

### Delphi survey

The initial assessment and harmonisation of ICATUS activities were reviewed independently and anonymously by all content experts as part of a Delphi decisional process. The Delphi method consists of a series of anonymous surveys, conducted to achieve a consensus among members of an expert panel, and it is widely used for decision-making [66]. The Delphi survey was conducted using Qualtrics software (Version qualtrics^XM^ of the Qualtrics Research Suite, Qualtrics LLC, Provo, UT, USA), an online survey platform [67]. Content experts were grouped into four panels, each consisting of four members. Each panel reviewed approximately 130 activities. Each panel included: i) the initial assessor (NL), who could provide detailed reasoning for every assessment to the other members of the panel; ii) at least one specialist in SB and/or PA epidemiology; iii) at least one specialist in SB and/or PA measurement; iv) at least one specialist in time-use surveys; and v) researchers from three or more different countries. The Delphi process was moderated by a researcher specialised in SB and PA topics, who was not involved in any of the Delphi panels nor was included in the author team.

At the beginning of the Delphi survey, panellists were given detailed information about the process of classifying the ICATUS activities by METs, wakefulness status, and posture. As part of the survey, each expert panel was asked to review the initial assessments and harmonisation and to express their agreement or provide suggestions for improvement. After each survey round, the moderator summarised the responses from the expert panels and amended the assessments and harmonisation accordingly. The revised list was then circulated among the members of the expert panel as part of the following survey round, to see if any further refinements were needed. A summary report including the original responses from all panel members was sent alongside all subsequent surveys. These steps were repeated until a consensus was reached among all content experts.

An additional panel was formed to review 32 ICATUS 2016 activities that could not be harmonised with a single activity from the Trial ICATUS 2005. We undertook the same Delphi procedures for this additional expert panel as described above.

## Results

**Fig. 1:**
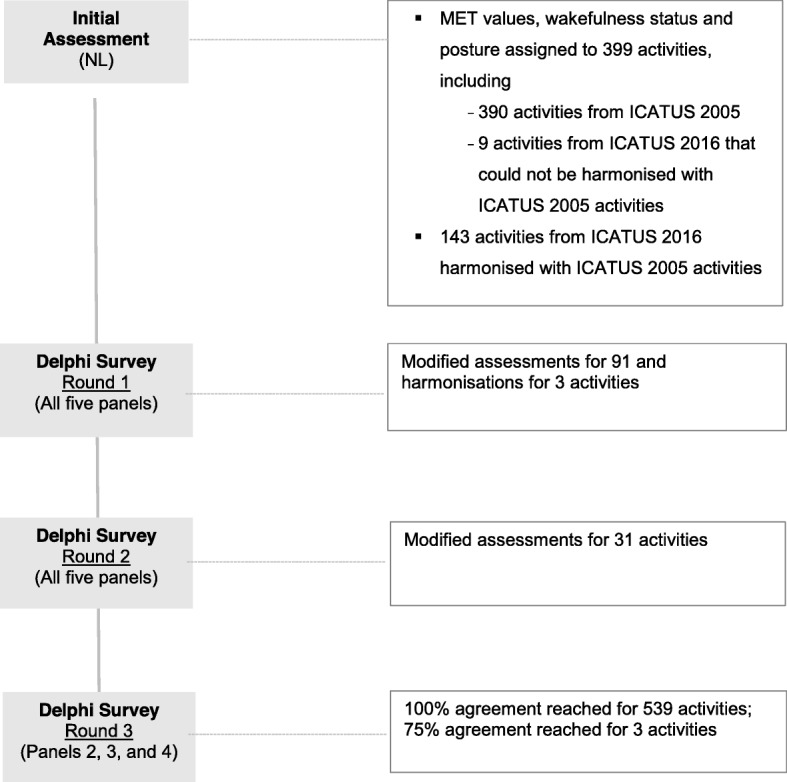
Flow and results of the Delphi process

We assigned MET estimates and codes for wakefulness status and posture to a total of 542 ICATUS activities. In Round 1, experts suggested modifying the original assessments of 91 activities and harmonisation of 3 activities. In Round 2, a consensus on the assessment and harmonisation of ICATUS 2005 and ICATUS 2016 activities was reached by two panels. Further suggestions were received to adjust assessments of 31 activities in the remaining groups. In Round 3, a consensus on the assessment and harmonisation of ICATUS 2005 and ICATUS 2016 activities was reached for the remaining groups. The experts reached consensus for all activities, except for: 131120 *“biking, skating, skateboarding”*; 131150 *“ball games, team sports”*; and 131160 *“water sports”*. These activities were assigned 7 METs, 7 METs, and 6 METs, respectively; however, one panel member suggested their metabolic values may be higher. For these activities, we made the final decisions in the third round of the Delphi survey, based on 75% agreement between the experts. The flow of the Delphi process and results of each survey round are outlined in Fig. 1.

From a total of 390 activities assessed from the Trial ICATUS 2005, we classified 3 activities into sleep (0.7%), 65 activities into SB (16.7%), 186 activities into LPA (47.7%), and 136 activities into MVPA (34.9%). The summary codes, including the activity-based categories, MET estimates, wakefulness status, and posture assigned to the Trial ICATUS 2005 activities are available in Additional file 2.

Of a total of 152 activities assessed from the final ICATUS 2016, we classified 3 activities into sleep (2%), 32 activities into SB (21%), 69 activities into LPA (45.4%), and 48 activities into MVPA (31.6%). We were able to harmonise a vast majority of ICATUS 2016 activities with ICATUS 2005 activities (*n* = 143; 94.1%). The summary codes, including the movement categories, MET estimates, wakefulness status, and posture assigned to the ICATUS 2016 activities are provided in Additional file 3. The full assessment and harmonisation tables of ICATUS activities are available in Additional file 1.

## Discussion

This is the first study to develop an expert-based classification of ICATUS activities into sleep, SB, LPA, and MVPA categories. We also provided estimated MET values, wakefulness status and posture for ICATUS activities; information that researchers can use for other categorisations (e.g., sleep, SB, LPA, and moderate-vigorous PA). The classification may be considered as the first step towards greater utilisation of ICATUS-based time-use surveys in time-use epidemiology.

To date, it seems that only time-use surveys conducted in high-income countries have been used to estimate SB and PA levels. This includes studies based on ATUS [25, 27, 31, 68–71], American Heritage Time Use Study (AHTUS) [32, 72], GSS-TU [33, 35, 58, 64, 73, 74], Australian Time Use Survey [29, 30, 60, 61], the United Kingdom Time Use Survey [36], Belgian Time Use Survey (using HETUS classification) [51, 59], Multinational Time Use Study (MTUS) [52, 53], Dutch Time Use Survey [75], and Halifax Space-Time Activity Research survey (conducted in Halifax, Nova Scotia, Canada) [57]. To the best of our knowledge, no such studies have been conducted in low- and middle-income countries. ICATUS-based time-use surveys have been conducted in many low-, middle-, and high-income countries [22, 56]. Our results will enable easier utilisation of these abundant data for the purpose of studies in time-use epidemiology. However, more validation studies of time-use surveys for assessing SB and PA are still needed, especially in larger samples and against device-based measures of these behaviours.

It has been suggested that three rounds of Delphi surveys are sufficient to gather key feedback from the panel members [66, 76]. Further rounds are unlikely to provide additional essential information [66, 76]. Percent of agreement between experts in Delphi studies varies from as low as 55 to 100% [77]. In the present study, the panel members reached perfect agreement for nearly all activities in no more than three survey rounds. This indicates that the assignment of MET values, wakefulness status, and posture to ICATUS-based time-use categories was relatively straightforward. However, a number of points were raised by experts during the Delphi process, which shows the importance of using a collective (vs individual) approach when developing criteria for classifying time use into activity-based categories. It is possible that more rounds of Delphi surveys would be needed, if the panels included additional members. On the other hand, a large number of points to assess (as in the current study) generally makes reaching consensus more difficult.

Historically, time-use surveys were designed to capture a population’s time budget reflecting on social and economic perspectives such as labour force, unpaid work, work life balance, and gender equality [55]. Estimating MET values for some ICATUS activities was impossible or very challenging. Firstly, there are several broad categories in ICATUS that consist of a wide range of different activities. It was difficult to assign a specific MET value to such categories. For example, the activity 131110 *“walking and hiking; jogging and running”* under group 1311 *“participating in sports”* includes four main activities; namely, walking, hiking, jogging, and running, that can be associated with varying intensities ranging from 3.0 METs (Compendium code 17170 *“walking, 2.5 mph, level, firm surface”*) to 23 METs (Compendium code 12135 *“running, 14 mph (4.3 min/mile)”*) [63]. Secondly, assigning METs to ICATUS activities in the *“working time in formal sector employment”* (Major division 01 employment) and the travel-related activities was not possible due to insufficient information about these activities. In ICATUS, these activities are classified generally as “working time” and “travel-related” activities. For example, ICATUS code 011110 is defined as “working time in main job”. It is obvious that “working time” defined in such an unspecific way can include any type of work, which can be completely sedentary or extremely physically demanding. Similarly, “travel-related activities” can include any kind of transport, including its active (e.g., cycling) and passive (e.g., going by train) modes. In the current study, these activities were, therefore, coded as “not applicable”. However, for future users of ICATUS-based time-use data, it may be possible to estimate associated METs of these activities, if the participants’ responses are linked with additional, more specific questions about their occupation and modes of travel [23]. Such additional questions are often included in time-use surveys [23]. Once these variables are linked, MET estimates can be assigned using the Compendium [63] or from summary MET values previously assigned to a list of occupations [23, 25, 26, 58]. Similar difficulties were also reported in previous studies by Tudor-Locke et al. [25] and Spinney et al. [58].

There are several strengths of the current study. Firstly, the Delphi panellists were purposefully selected to participate in the study based on their expertise in relevant research fields. Secondly, Delphi panels were formed in a way to ensure representation of varying skills and experience in each panel. Thirdly, we categorised both ICATUS 2005 and ICATUS 2016 activities, which will enable SB and PA researchers to use ICATUS-based time-use data collected over a period of nearly 15 years. Lastly, our harmonisation of ICATUS 2005 and ICATUS 2016 activities will improve the comparability of the derived SB and PA data from the two ICATUS versions.

There are also some limitations in the present study. First, as we needed experts with relevant knowledge in different fields, we included 13 content experts to participate in the Delphi survey. As they were divided into four experts per one Delphi panel, the number of Delphi panellists in this study may be considered small. Despite our effort to recruit panellists with expertise in different areas, it is possible that their consensus does not represent the broader field. It may also be that the relatively small number of panel members negatively impacted the validity of final outcomes of the Delphi process. Another limitation of the study is that we assigned an unweighted median MET value to most ICATUS activities, calculated from the list of matched Compendium activities. A more precise estimation could be achieved by calculating weighted averages, where the weights are proportional to the representation of these activities in the time use of a specific population. This approach has been used with data from the MTUS [53], but it depends on an underlying dataset giving the prevalence of component activities. Given that we did not have access to such data as part of this study and that our study was not intended to focus on a specific population, we provided generic, non-weighted estimates. Furthermore, the MET values we used from the Compendium quantify energy costs of physical activities in healthy, 18–65 year old adults [63]. The MET values applied to ICATUS activities should not be interchanged with those identified in the Compendium. Therefore, our estimates are only applicable to healthy adults for analysis of ICATUS data. Detailed tables, including the lists of matched activities from the Compendium and calculations of summary METs are available in Additional file 1, if any adaptations to a specific population is required in future studies.

## Conclusion

In this study, a group of 13 content experts in measurement, epidemiology and time use reached a consensus about the estimated MET values, wakefulness status and posture of ICATUS 2005 and ICATUS 2016 activities. This has enabled categorisation of ICATUS activities into sleep, SB, LPA, and MVPA categories, which may encourage greater utilisation of data from time-use surveys in public health research. The generic estimates and categorisations we provided may be used or further adapted to better reflect the time-use patterns of specific study populations. Future research needs to assess the validity and reliability of SB and PA estimates from ICATUS-based time-use surveys. Provided the measurement properties are adequate, the new categorisation system can then be used in studies exploring the patterns, trends, determinants, and outcomes of sleep, SB, LPA, and MVPA.

## Supplementary information

**Additional file 1.** ICATUS Assessment and Harmonisation Tables. The full assessment and harmonisation tables of ICATUS activities.

**Additional file 2.** 2005 ICATUS Assignment Table. Metabolic equivalent (MET) values, summary codes and movement categories assigned to 2005 International Classification of Activities for Time-Use Statistics (ICATUS) activities.

**Additional file 3.** 2016 ICATUS Assignment Table. Metabolic equivalent (MET) values, summary codes and movement categories assigned to 2016 Inte.rnational Classification of Activities for Time-Use Statistics (ICATUS) activities.

## Data Availability

The assessment of ICATUS activities is available in Tables, Figure, and Supplementary materials.

## Abbreviations

AHTUS: American Heritage Time Use Study
ATUS: American Time Use Survey
GSS-TU: Statistics Canada’s General Social Survey – Time Use
HETUS: Harmonised European Time Use Survey
ICATUS: International Classification of Activities for Time-Use Statistics
ISCO: International Standard Classification of Occupations
LPA: Light Physical Activity
MET: Metabolic Equivalent of Task
MTUS: Multinational Time Use Study
MVPA: Moderate-to-Vigorous Physical Activity
PA: Physical Activity
SB: Sedentary Behaviour
SIC: Standard Industry Codes
SOC: Standard Occupational Classification
TOPAQ: Tecumseh Occupational Physical Activity Questionnaire
UNSD: United Nations Statistics Division
VIRTUE: Viable Integrative Research in Time-Use Epidemiology
