# Supplementary material for: Standardised criteria for classifying the International Classification of Activities for Time-use Statistics (ICATUS) activity groups into sleep, sedentary behaviour, and physical activity
Source: Int J Behav Nutr Phys Act. 2019 Nov 14;16:106. doi: 10.1186/s12966-019-0875-5 (PMC6857154; doi:10.1186/s12966-019-0875-5)
Supplement: Supplementary file 2 — Additional file 2. 2005 ICATUS Assignment Table. Metabolic equivalent (MET) values, summary codes and movement categories assigned to 2005 International Classification of Activities for Time-Use Statistics (ICATUS) activities. [file 12966_2019_875_MOESM2_ESM.pdf]

Metabolic equivalent (MET) values, summary codes and movement categories assigned to

International Classification of Activities for Time Use Statistics (ICATUS) 2005 activities

ICATUS 2005 activity				Category	MET	Wakeful-	Sitting/
Code			Title			ness	lying
0111			**Working time in "formal sector" employment**				
	01111	011110	Working time in main job	n/a	n/a	n/a	n/a
	01112	011120	Working time in other jobs	n/a	n/a	n/a	n/a
	01113	011130	Working time as apprentice, intern and related positions	n/a	n/a	n/a	n/a
	01114	011140	Short breaks and interruptions from work	SB	1.30	yes	yes
	01115	011150	Training and studies in relation to work in the "formal sector"	LPA	1.80	yes	yes
	0111x		Working time in "formal sector" employment n.f.d.	n/a	n/a	n/a	n/a
0112			**Other breaks**				
	01121	011210	Idle time before/after work	SB	1.30	yes	yes
	01122	011220	Lunch break from work	SB	1.50	yes	yes
0120			**Looking for work/setting up business in the "formal sector"**				
	01201	012010	Looking for work in the "formal sector"	SB	1.40	yes	yes
	01202	012020	Looking for/setting up business in the "formal sector"	LPA	1.65	yes	yes
0130			**Travel related to work in the "formal sector"**				
	01300	013000	Travel related to work in the "formal sector"	n/a	n/a	n/a	n/a
0190			**Work in the "formal sector" n.e.c.**				
	01900	019000	Work in the "formal sector" n.e.c.	n/a	n/a	n/a	n/a
0211			**Working time in primary production activities**				
	02111		Growing of crops and trees; kitchen gardening	MVPA	3.55	yes	no
		021111	Land preparation	MVPA	5.40	yes	no
		021112	Sowing and planting operations	MVPA	4.30	yes	no
		021113	Collecting and preparing organic fertilizer, carrying and spreading organic/chemical fertilizer	MVPA	3.15	yes	no
		021114	Field/garden upkeep	MVPA	3.90	yes	no
		021115	Harvesting	MVPA	3.55	yes	no
		021116	Post-harvest activities	MVPA	3.50	yes	no
		021117	Other agricultural service activities	MVPA	3.50	yes	no
		021119	Other specified activities related to growing of crops and trees	MVPA	3.55	yes	no
		02111x	Growing of crops and trees; kitchen gardening n.f.d.	MVPA	3.55	yes	no
	02112		Farming of animals; production of animal products; animal husbandry services	MVPA	4.30	yes	no
		021121	Fodder collection; preparation of feed; feeding, watering; grazing	MVPA	4.30	yes	no
		021122	Grooming, shoeing, cleaning; veterinary care	MVPA	4.50	yes	no
		021123	Washing shed, coop cleaning	MVPA	4.80	yes	no
		021124	Work related to breeding; hatching	MVPA	4.65	yes	no
		021125	Milking and processing of raw milk	LPA	2.40	yes	no
		021126	Collecting, storing, grading of eggs	MVPA	3.30	yes	no
		021127	Shearing, producing hides and skins from ranching	MVPA	4.00	yes	no
		021128	Dung-gathering and making dung cakes	MVPA	4.30	yes	no

ICATUS 2005 activity Code		Title	Category	MET	Wakeful-ness	Sitting/lying
	021129	Other specified activities related to animal farming, production of animal products, animal husbandry services	MVPA	4.30	yes	no
	02112x	Farming of animals; production of animal products; animal husbandry services n.f.d.	MVPA	4.30	yes	no
02113		Hunting, trapping and production of animal skins	MVPA	3.30	yes	no
	021131	Hunting and trapping wild animals	LPA	2.50	yes	no
	021132	Hunting birds	MVPA	3.30	yes	no
	021133	Production of fur skins, reptile or bird skins from hunting and trapping	MVPA	3.50	yes	no
	021139	Other specified activities related to hunting and production of animal skins	MVPA	3.30	yes	no
	02113x	Hunting, trapping and production of animal skins n.f.d.	MVPA	3.30	yes	no
02114		Gathering of wild products, woodcutting, gathering firewood and other forestry activities	MVPA	3.90	yes	no
	021141	Gathering medicinal and other plants for craft production or fuel	MVPA	3.50	yes	no
	021142	Gathering wild fruits, berries or other uncultivated crops, other edible food	MVPA	3.50	yes	no
	021143	Woodcutting and gathering firewood	MVPA	5.30	yes	no
	021144	Reforestation, growing forest trees, replanting	MVPA	4.30	yes	no
	021149	Other specified activities related to hunting, forestry, and gathering of wild products	MVPA	3.90	yes	no
	02114x	Gathering of wild products, woodcutting, gathering firewood and other forestry activities n.f.d.	MVPA	3.90	yes	no
02115		Fishing and fish/aquatic farming	MVPA	4.15	yes	no
	021151	Catching fish and gathering other forms of aquatic life	MVPA	4.00	yes	no
	021152	Gathering marine materials such as natural pearls, sponges, corals, algae, seashells	MVPA	6.00	yes	no
	021153	Fish/aquatic farming: breeding, rearing	MVPA	4.65	yes	no
	021154	Fish/aquatic farming: cleaning beds, feeding	MVPA	4.15	yes	no
	021155	Repair, care and maintenance of fishing boats and equipment, tools, fishnets	MVPA	3.50	yes	no
	021159	Other specified activities related to fishing, fish/aquatic farming	MVPA	4.15	yes	no
	02115x	Fishing and fish/aquatic farming n.f.d.	MVPA	4.15	yes	no
02116		Mining and quarrying	MVPA	5.50	yes	no
	021161	Mining/extraction of salt	MVPA	5.50	yes	no
	021162	Drilling well, boring holes etc	MVPA	5.80	yes	no
	021163	Quarrying of stone slabs	MVPA	5.50	yes	no
	021164	Crushing and breaking of stones	MVPA	6.00	yes	no
	021165	Digging out clay, gravel and sand	MVPA	5.90	yes	no
	021166	Gold panning, mining gems etc.	MVPA	5.50	yes	no
	021167	Transporting, storing and stocking	MVPA	3.00	yes	no
	021169	Other specified mining and quarrying activities	MVPA	5.50	yes	no
	02116x	Mining and quarrying n.f.d.	MVPA	5.50	yes	no
02117	021170	Collecting water	MVPA	4.30	yes	no
02118	021180	Training and studies in relation to work in primary production activities of households	LPA	2.40	yes	yes

Code			ICATUS 2005 activity Title	Category	MET	Wakeful-ness	Sitting/ lying
	0211x		Working time in primary production activities n.f.d.	MVPA	4.03	yes	no
0212			**Working time in primary production activities**				
	02121	021210	Purchasing/acquiring inputs/supplies used for primary production activities of households	LPA	2.05	yes	no
	02122	021220	Selling/disposing of outputs of primary production activities of households	LPA	2.00	yes	no
0220			**Looking for work/setting up business in household primary production activities**				
	02201	022010	Looking for work in primary production activities in household enterprise	SB	1.50	yes	yes
	02202	022020	Looking for/setting up business in primary production activities in household enterprise	LPA	1.65	yes	yes
0230			**Travel related to primary production activities of households**				
	02300	023000	Travel related to primary production activities of households	n/a	n/a	n/a	n/a
0290			**Work for households in primary production activities n.e.c.**				
	02900	029000	Work for households in primary production activities n.e.c.	MVPA	3.55	yes	no
0311			**Working time in non-primary production activities**				
	03111		Processing of food products	LPA	2.50	yes	no
		031111	Production, processing and preserving of meat and meat products	MVPA	3.00	yes	no
		031112	Making dairy products	LPA	2.00	yes	no
		031113	Processing and preserving of fish and fish products	LPA	2.30	yes	no
		031114	Processing and preserving of fruits and vegetables	LPA	2.50	yes	no
		031115	Processing grains	LPA	2.90	yes	no
		031119	Other specified activities related to processing of food products	LPA	2.50	yes	no
		03111x	Processing of food products n.f.d.	LPA	2.50	yes	no
	03112		Making of other food products and beverages	LPA	2.83	yes	no
		031121	Beer brewing and making of other beverages, wines or spirits	MVPA	3.25	yes	no
		031122	Baking bread, cakes, rice cakes, pastries, pies, tarts, biscuits	MVPA	3.00	yes	no
		031123	Making noodles, pasta and similar products	MVPA	3.00	yes	no
		031124	Making candy, boiled sweets, caramel, chocolate, and other sugar confectionery products	LPA	2.65	yes	no
		031125	Roasting seeds, nuts	LPA	2.00	yes	no
		031126	Roasting, grinding coffee beans	LPA	2.15	yes	no
		031129	Other specified activities related to making of other food products and beverages	LPA	2.83	yes	no
		03112x	Making of other food products and beverages n.f.d.	LPA	2.83	yes	no
	03113		Making textiles, wearing apparel, leather and associated products	MVPA	3.00	yes	yes
		031131	Spinning, weaving, finishing of textiles	MVPA	3.00	yes	yes
		031132	Producing articles from textile except apparel	LPA	2.00	yes	yes
		031133	Making wearing apparel	LPA	2.40	yes	yes
		031134	Curing of skins and production of leather, tanning and dressing of leather	MVPA	3.50	yes	no
		031135	Making shoes, footwear, handbags, luggage	MVPA	3.00	yes	yes

Code			ICATUS 2005 activity Title	Category	MET	Wakeful-ness	Sitting/lying
		031139	Other specified activities related to making textiles, wearing apparel, leather and associated products	MVPA	3.00	yes	yes
		03113x	Making textiles, wearing apparel, leather and associated products n.f.d.	MVPA	3.00	yes	yes
	03114		Craft-making using all types of materials	MVPA	3.00	yes	yes
		031141	Making wood products including furniture, fixtures or furnishings, statuettes and other ornaments	MVPA	3.30	yes	no
		031142	Making baskets, wickerwork and other similar products	MVPA	3.00	yes	yes
		031143	Fabricating utensils, cutlery, hand tools and other metal products	MVPA	3.00	yes	no
		031144	Metal working	MVPA	4.50	yes	no
		031145	Making pottery, ovens and cooking stoves, ornaments etc. from clay, plaster or cement	MVPA	3.00	yes	yes
		031146	Making paper and paper products; paper crafts	LPA	2.05	yes	yes
		031147	Making soap, perfume, candles etc.	MVPA	3.25	yes	no
		031149	Other specified activities related to craft-making	MVPA	3.00	yes	yes
		03114x	Craft-making using all types of materials n.f.d.	MVPA	3.00	yes	yes
	03115	031150	Tobacco preparing and curing	MVPA	3.00	yes	no
	03116	031160	Making bricks, concrete slabs, hollow blocks, tiles etc.	MVPA	4.75	yes	no
	03117	031170	Making herbal and medicinal preparations	SB	1.40	yes	yes
	03118	031180	Training and studies in relation to work in non-primary production activities of households	LPA	2.40	yes	yes
	0311x		Working time in non-primary production activities n.f.d	LPA	2.91	yes	no
0312			Acquiring inputs/supplies and disposing of outputs used for non-primary production activities households				
	03121	031210	Purchasing/acquiring inputs/supplies used for non-primary production activities for households	LPA	2.05	yes	no
	03122	031220	Selling/disposing of outputs of non-primary production activities of households	LPA	2.00	yes	no
0320			Looking for work/setting up business in non-primary production activities in household enterprise				
	03201	032010	Looking for work in non-primary production activities in household enterprise	SB	1.50	yes	yes
	03202	032020	Looking for/setting up business in non-primary production activities in household enterprise	LPA	1.65	yes	yes
0330			Travel related to non-primary production of household				
	03300	033000	Travel related to non-primary production of household	n/a	n/a	n/a	n/a
0390			Work for household in non-primary production activities n.e.c.				
	03900	039000	Work for household in non-primary production activities n.e.c.	LPA	2.50	yes	no
0411			Working time in construction activities				
	04111		Construction and repair for own capital formation	MVPA	4.30	yes	no
		041111	Building of own house	MVPA	4.30	yes	no
		041112	Major home improvements and repairs	MVPA	4.00	yes	no
		041113	Building and repair of animal and poultry sheds/shelter, business place, field walls/fences, storage facilities for farm produce, irrigation	MVPA	4.30	yes	no

ICATUS 2005 activity				Category	MET	Wakeful-ness	Sitting/lying
Code			Title				
		041119	Other specified activities related to construction and repair for own capital formation	MVPA	4.30	yes	no
		04111x	Construction and repair for own capital formation n.f.d.	MVPA	4.30	yes	no
	04112	041120	Construction and repair of buildings, roads, dams and other structures	MVPA	4.50	yes	no
	04113	041130	Community-organized construction and major repairs of roads, buildings, bridges, dams etc.	MVPA	4.50	yes	no
	04114	041140	Training and studies in relation to work in construction activities in household enterprise	MVPA	3.25	yes	no
	0411x		Working time in construction activities n.f.d.	MVPA	4.40	yes	no
0412			Acquiring inputs/supplies for construction activities for household production				
	04120	041200	Purchasing/acquiring inputs/supplies for construction activities for household production	LPA	1.80	yes	yes
0420			Looking for work/setting up business in construction activities in household enterprise				
	04201	042010	Looking for work in construction activities in household enterprise	SB	1.50	yes	yes
	04202	042020	Looking for/setting up business in construction activities as household enterprise	LPA	1.65	yes	yes
0430			Travel related to construction activities of households				
	04300	043000	Travel related to construction activities of households	n/a	n/a	n/a	n/a
0490			Work for household in construction activities n.e.c.				
	04900	049000	Work for household in construction activities n.e.c.	MVPA	3.78	yes	no
0511			Food vending and trading				
	05111		Preparing and selling food and beverage	MVPA	3.00	yes	no
		051111	Preparing/packing food and beverage preparations	MVPA	3.00	yes	no
		051112	Selling/delivering food and beverage preparations	MVPA	3.00	yes	no
		051113	Putting up food stalls; cleaning and maintenance	LPA	2.50	yes	no
		051119	Other specified activities related to preparing and selling food and beverage	MVPA	3.00	yes	no
		05111x	Preparing and selling food and beverage n.f.d.	MVPA	3.00	yes	no
	05112		Petty trading, door-to-door vending, street vending, hawking	LPA	2.00	yes	no
		051121	Petty trading	LPA	2.00	yes	no
		051122	Door-to-door vending	MVPA	3.50	yes	no
		051123	Street vending, hawking and other itinerant trading	LPA	1.90	yes	no
		051129	Other specified activities related to petty trading and vending activities	LPA	2.00	yes	no
		05112x	Trading n.f.d.	LPA	2.00	yes	no
0512			Providing repair, installation and maintenance services				
	05121	051210	Fitting, installing, tool setting, maintaining and repairing tools and machinery	MVPA	3.00	yes	no
	05122	051220	Repair of vehicles	MVPA	3.65	yes	no
	05123	051230	Repair of personal goods	LPA	2.30	yes	no
	05124	051240	Repair of household goods	MVPA	3.00	yes	no

ICATUS 2005 activity Code			Title	Category	MET	Wakeful-ness	Sitting/ lying
0513			Providing business and professional services				
	05131	051310	Renting out rooms, sleeping space and associated work	LPA	1.65	yes	yes
	05132	051320	Lending and collecting money; foreign exchange	SB	1.50	yes	yes
	05133	051330	Typing, word-processing, programming, encoding	SB	1.30	yes	yes
	05134	051340	Accounting, bookkeeping, legal and related services	SB	1.30	yes	yes
	05135	051350	Tutoring	LPA	1.65	yes	yes
	05136	051360	Provision of medical and dental services	LPA	2.00	yes	no
	05137	051370	Provision of nursing/therapy services	LPA	2.00	yes	no
0514			Providing personal care services				
	05141	051410	Provision of personal care services	LPA	1.80	yes	no
	05142	051420	Provision of non-professional health-care	LPA	2.00	yes	no
0515			Transporting goods and passengers				
	05151	051510	Transporting goods	LPA	2.50	yes	yes
	05152	051520	Transporting passengers	LPA	2.50	yes	yes
0516			Paid domestic services				
	05160	051600	Providing paid domestic services	LPA	2.23	yes	no
0517			Meetings/training and studies				
	05170	051700	Training and studies related to work in service activities	LPA	1.65	yes	yes
0520			Looking for work/setting up business in service activities in household enterprise				
	05200	052000	Looking for work in service activities in household enterprise	SB	1.50	yes	yes
0530			Travel related to providing services for income				
	05300	053000	Travel related to providing services for income	n/a	n/a	n/a	n/a
0590			Work for household providing services for income n.e.c.				
	05900	059000	Work for household providing services for income n.e.c.	LPA	2.00	yes	no
0611			Unpaid domestic services				
	06111		Food management	LPA	2.50	yes	no
		061111	Preparing meals/snacks	LPA	2.50	yes	no
		061112	Serving meals/snacks	LPA	2.50	yes	no
		061113	Cleaning up after food preparation/meals/snacks	LPA	2.50	yes	no
		061119	Other specified activities related to food management	LPA	2.50	yes	no
		06111x	Food management n.f.d.	LPA	2.50	yes	no
	06112		Cleaning and upkeep of dwelling and surroundings	MVPA	3.35	yes	no
		061121	Indoor cleaning	MVPA	3.30	yes	no
		061122	Outdoor cleaning	MVPA	4.00	yes	no
		061123	Recycling; disposal of garbage	LPA	2.50	yes	no
		061124	Care of outdoor garden, landscaping, trimming, grounds/yard/lawn maintenance	MVPA	4.15	yes	no
		061125	Heating and water supply (including tending furnaces, boilers and fire places)	MVPA	3.40	yes	no
		061126	Making various household arrangements	LPA	2.50	yes	no
		061129	Other specified activities related to cleaning and upkeep of dwelling and surroundings	MVPA	3.35	yes	no
		06112x	Cleaning and upkeep of dwelling and surroundings n.f.d.	MVPA	3.35	yes	no

ICATUS 2005 activity			Category	MET	Wakeful-ness	Sitting/ lying
Code		Title				
	06113	Do-it-yourself decoration, maintenance and small repairs	MVPA	3.00	yes	no
	061131	Do-it-yourself improvement, maintenance and repair of dwellings	MVPA	3.30	yes	no
	061132	Installation, servicing and repair of personal and household goods	MVPA	3.00	yes	no
	061133	Vehicle maintenance and minor repairs	LPA	2.65	yes	no
	061139	Other specified activities related to do-it-yourself decoration, maintenance and small repairs	MVPA	3.00	yes	no
	06113x	Do-it-yourself decoration, maintenance and small repairs n.f.d.	MVPA	3.00	yes	no
	06114	Care of textiles and footwear	LPA	2.40	yes	no
	061141	Hand-washing; loading/unloading washing machine	MVPA	3.00	yes	no
	061142	Drying; hanging out, bringing in wash	MVPA	3.00	yes	no
	061143	Ironing/pressing	LPA	1.80	yes	yes
	061144	Sorting, folding, storing	LPA	2.15	yes	no
	061145	Mending/repairing and care of clothes; cleaning and polishing shoes	LPA	2.40	yes	yes
	061149	Other specified care of textiles and footwear	LPA	2.40	yes	no
	06114x	Care of textiles and footwear n.f.d.	LPA	2.40	yes	no
	06115	Household management	LPA	2.30	yes	yes
	061151	Paying household bills (utilities, cable television etc.)	LPA	2.30	yes	yes
	061152	Budgeting, organizing, planning	SB	1.50	yes	yes
	061153	Selling, disposing of household assets	LPA	2.30	yes	yes
	061159	Other specified household management	LPA	2.30	yes	yes
	06115x	Household management n.f.d.	LPA	2.30	yes	yes
	06116	Pet care	LPA	2.53	yes	no
	061161	Daily care including feeding, cleaning, grooming, walking	LPA	2.75	yes	no
	061162	Taking pets for veterinary care	LPA	2.30	yes	no
	061169	Other specified pet care	LPA	2.53	yes	no
	06116x	Pet care n.f.d.	LPA	2.53	yes	no
0612		Shopping				
	06121	Shopping for/purchasing of goods and related activities	LPA	2.00	yes	no
	061211	Shopping for/purchasing of consumer goods	LPA	2.30	yes	no
	061212	Shopping for/purchasing of durable/capital goods	LPA	1.80	yes	no
	061213	Window shopping	LPA	2.00	yes	no
	061219	Other specified shopping for/purchasing of goods and related activities	LPA	2.00	yes	no
	06121x	Shopping for/purchasing of goods and related activities n.f.d.	LPA	2.00	yes	no
	06122	Shopping for/availing of services and related activities	LPA	1.65	yes	yes
	061221	Shopping for/availing of repair and maintenance services	LPA	1.80	yes	yes
	061222	Shopping for/availing of administrative services	LPA	1.65	yes	yes
	061223	Shopping for personal care services (not for oneself)	LPA	1.65	yes	yes
	061224	Shopping for medical and health-care services (not for oneself)	LPA	1.65	yes	yes
	061225	Shopping for/availing of childcare services	LPA	1.65	yes	yes
	061226	Shopping for educational services	LPA	1.65	yes	yes
	061229	Other specified shopping/availing of services	LPA	1.65	yes	yes

ICATUS 2005 activity Code			Title	Category	MET	Wakeful-ness	Sitting/ lying
		06122x	Shopping for/availing of services and related activities n.f.d	LPA	1.65	yes	yes
0620			Travel related to provision of unpaid domestic services				
	06200	062000	Travel related to provision of unpaid domestic services	n/a	n/a	n/a	n/a
0690			Unpaid domestic services n.e.c.				
	06900	069000	Unpaid domestic services n.e.c.	LPA	2.45	yes	no
0711			childcare				
	07111		Caring for children/physical care	LPA	2.00	yes	no
		071111	General childcare	LPA	2.15	yes	no
		071112	Putting children to bed	SB	1.40	yes	yes
		071113	Getting children ready for school	LPA	2.50	yes	no
		071114	Giving personal care to children	LPA	2.00	yes	no
		071115	Giving medical/health-care to children	LPA	2.00	yes	no
		071119	Other specified physical care of children	LPA	2.00	yes	no
		07111x	Caring for children/physical care n.f.d.	LPA	2.00	yes	no
	07112		Teaching, training, helping children	LPA	2.20	yes	no
		071121	Teaching children	MVPA	3.00	yes	no
		071122	Reading, playing and talking with children	LPA	2.20	yes	no
		071123	Giving emotional support to children	LPA	1.75	yes	yes
		071129	Other specified teaching, training, helping activities	LPA	2.20	yes	no
	07113		Accompanying children to places	LPA	1.80	yes	no
		071131	Accompanying children to receive personal services	LPA	1.80	yes	no
		071132	Accompanying children to receive medical/health services	LPA	1.80	yes	no
		071133	Accompanying children to school, day-care centres	LPA	1.80	yes	no
		071134	Accompanying children to sports, lessons etc.	LPA	1.80	yes	no
		071135	Taking children on excursions, museum visits and similar outings; coordinating or facilitating child's social or non-school activities	LPA	2.00	yes	no
		071139	Accompanying children to other specified places	LPA	1.80	yes	no
		07113x	Accompanying children to places n.f.d.	LPA	1.80	yes	no
	07114	071140	Minding children (passive care)	SB	1.30	yes	yes
0712			Adult care				
	07121		Caring for adults/physical care	MVPA	3.00	yes	no
		071211	Giving personal care to adults	MVPA	3.00	yes	no
		071212	Giving medical/health-care to adults	MVPA	3.00	yes	no
		071219	Other specified physical care of adults	MVPA	3.00	yes	no
		07121x	Caring for adults/physical care n.f.d.	MVPA	3.00	yes	no
	07122	071220	Caring for adults/emotional support	SB	1.50	yes	yes
	07123		Accompanying adults to places	LPA	2.40	yes	no
		071231	Accompanying adults to receive personal services	LPA	2.50	yes	no
		071232	Accompanying adults to receive medical/health services	LPA	2.50	yes	no
		071233	Accompanying adults for shopping	LPA	2.40	yes	no
		071234	Accompanying adults to social activities	LPA	2.25	yes	no
		071235	Accompanying adults to cultural, sports and entertainment venues	LPA	2.25	yes	no
		071239	Accompanying adults to other specified places	LPA	2.40	yes	no

ICATUS 2005 activity Code			Title	Category	MET	Wakeful-ness	Sitting/ lying
		07123x	Accompanying adults to places n.f.d.	LPA	2.40	yes	no
0720			Travel related to unpaid caregiving services to household members				
	07200	072000	Travel related to unpaid caregiving services to household members	n/a	n/a	n/a	n/a
0790			Providing unpaid caregiving services to household members n.e.c.				
	07900	079000	Providing unpaid caregiving services to household members n.e.c.	LPA	2.00	yes	no
0811			Unpaid help to other households				
	08111		Household maintenance and management as help to other households	LPA	2.40	yes	no
		081111	Preparing and serving meals as help to other households	LPA	2.50	yes	no
		081112	Cleaning and upkeep as help to other households	MVPA	3.30	yes	no
		081113	Care of textiles as help to other households	LPA	2.30	yes	no
		081114	Household management as help to other households	LPA	2.30	yes	no
		081115	Pet care as help to other households	LPA	2.40	yes	no
		081119	Other specified help to other households	LPA	2.40	yes	no
		08111x	Household maintenance and management as help to other households n.f.d.	LPA	2.40	yes	no
	08112		Shopping for/purchasing of goods and services as help to other households	LPA	2.05	yes	no
		081121	Shopping for/purchasing of goods as help	LPA	2.30	yes	no
		081122	Shopping for/purchasing of services as help	LPA	1.80	yes	no
		081129	Other specified shopping/purchasing as help	LPA	2.05	yes	no
		08112x	Shopping for/purchasing of goods and services as help to other households n.f.d.	LPA	2.05	yes	no
	08113	081130	Construction, renovation and repairs of dwellings and other structures as help to other households	MVPA	4.00	yes	no
	08114	081140	Repairs of consumer and household goods as help to other households	LPA	2.75	yes	no
	08115	081150	Unpaid help in business/farm and employment as help to other households	LPA	2.30	yes	no
	08116	081160	Childcare as help to other households	LPA	2.50	yes	no
	08117	081170	Adult care as help to other households	MVPA	3.15	yes	no
	08118	081180	Transportation assistance to other households	LPA	2.50	yes	yes
0812			Community-organized services				
	08121	081210	Community organized work: cooking for collective celebrations etc.	LPA	2.50	yes	no
	08122	081220	Work on road/building repair, clearing and preparing community land, cleaning (streets, markets etc.)	MVPA	4.75	yes	no
	08123	081230	Organizing and work on community-based assistance to villages, other sublocations	LPA	1.90	yes	yes
	08124	081240	Organizing and work on community-based assistance to families and individuals	LPA	1.90	yes	yes
0813			Organized unpaid volunteer services				

ICATUS 2005 activity Code			Title	Category	MET	Wakeful-ness	Sitting/ lying
	08131	081310	Volunteer work for organizations (not directly for individuals)	LPA	2.30	yes	yes
	08132	081320	Volunteer work through organizations (extended directly to individuals)	MVPA	3.00	yes	no
0820			Attendance in meetings				
	08200	082000	Attendance in meetings	SB	1.50	yes	yes
0830			Other community services				
	08300		Involvement in civic and related responsibilities	LPA	2.30	yes	no
		083001	Attending civic ceremonies	LPA	2.00	yes	no
		083002	Attending to civic obligations	LPA	2.30	yes	no
		083009	Other specified involvement in civic and related responsibilities	LPA	2.30	yes	no
		08300x	Involvement in civic and related responsibilities n.f.d.	LPA	2.30	yes	no
0840			Travel related to community services and help to other households				
	08400	084000	Travel related to community services and help to other households	n/a	n/a	n/a	n/a
0890			Community services and help to other households n.e.c.				
	08900	089000	Community services and help to other households n.e.c.	LPA	2.45	yes	no
0911			General education				
	09111		School/university attendance	LPA	2.15	yes	yes
		091111	Attending class/lecture including taking examinations	SB	1.30	yes	yes
		091112	Engaging in co-curricular and extra-curricular activities	MVPA	3.00	yes	no
		091119	Other specified activities related to school/university attendance	LPA	2.15	yes	yes
		09111x	School/university attendance n.f.d.	LPA	2.15	yes	yes
	09112	091120	Breaks/waiting at place of general education	LPA	1.65	yes	no
	09113	091130	Self-study for distance education course work (video, audio, online)	SB	1.30	yes	yes
0912			Homework, course review, research related to general education				
	09120	091200	Homework, course review, research related to general education	SB	1.40	yes	yes
0913			Additional study, non-formal education and courses during free time				
	09130	091300	Additional study, non-formal education and courses during free time	SB	1.40	yes	yes
0914			Career/professional development training and studies				
	09140	091400	Career/professional development training and studies	LPA	1.65	yes	yes
0920			Other activities carried out in relation to learning activities				
	09200	092000	Other activities carried out in relation to learning activities	LPA	2.00	yes	no
0930			Travel related to learning				
	09300	093000	Travel related to learning	n/a	n/a	n/a	n/a
0990			Learning activities n.e.c.				
	09900	099000	Learning activities n.e.c.	LPA	1.65	yes	yes
1011			Socializing and communication				
	10111		Talking, conversing	SB	1.50	yes	yes
		101111	Talking/conversing face to face	SB	1.50	yes	yes
		101112	Talking/conversing by telephone, texting, short-wave radio etc	SB	1.50	yes	yes

ICATUS 2005 activity				Category	MET	Wakeful-ness	Sitting/ lying
Code			Title				
		101113	Cyber-chatting including instant messaging, discussion groups etc.	SB	1.50	yes	yes
		101119	Other specified activities related to talking/conversing	SB	1.50	yes	yes
		10111x	Talking, conversing n.f.d.	SB	1.50	yes	yes
	10112		Socializing activities	LPA	1.90	yes	yes
		101121	Doing activities/going to places or events together	LPA	2.00	yes	no
		101122	Receiving visitors	LPA	1.80	yes	yes
		101123	Visiting friends and relatives	LPA	1.80	yes	yes
		101124	Hosting parties, receptions, similar gatherings	LPA	1.80	yes	yes
		101125	Attending parties, receptions, similar gatherings	LPA	2.00	yes	yes
		101126	Socializing at bars, clubs	LPA	2.50	yes	no
		101129	Other specified socializing activities	LPA	1.90	yes	yes
		10112x	Socializing n.f.d.	LPA	1.90	yes	yes
	10113	101130	Reading and writing mail	SB	1.30	yes	yes
	10114	101140	Unsocial/antisocial/negative social activities	LPA	1.80	yes	no
	1011x		Socializing and communication n.f.d.	LPA	1.65	yes	yes
1012			Participating in community cultural/social events				
	10121	101210	Participating in community celebrations of cultural/historic events	LPA	1.80	yes	no
	10122	101220	Participating in community rites/events (non-religious) of weddings, funerals, births and similar rites-of-passage	LPA	1.80	yes	no
	10123	101230	Participating in community social functions (music, dance etc.)	LPA	2.00	yes	no
	1012x		Community participation n.f.d.	LPA	1.80	yes	no
1020			Travel related to socializing and community participation				
	10200	102000	Travel related to socializing and community participation	n/a	n/a	n/a	n/a
1090			Socializing and community participation n.e.c.				
	10900	109000	Socializing and community participation n.e.c.	LPA	1.80	yes	yes
1111			Attendance at organized/mass cultural events				
	11111	111110	Visit museum, art gallery, historical/cultural park, heritage site	MVPA	3.50	yes	no
	11112	111120	Attendance at movies/cinema	SB	1.50	yes	yes
	11113	111130	Attendance at theatre, opera, ballet, concerts	LPA	1.80	yes	yes
	11119	111190	Attendance at other specified mass cultural events	MVPA	3.50	yes	no
1112			Attendance at parks/gardens, shows				
	11120	111200	Attendance/visit to zoo, animal park, botanic garden, amusement centre, fairs, festivals, circus, animal shows, plant shows	MVPA	3.30	yes	no
1113			Attendance at sports events				
	11131	111310	Attendance at professional sports events	LPA	2.40	yes	yes
	11132	111320	Attendance at amateur sports events	LPA	2.40	yes	yes
1120			Travel related to attending/visiting cultural, entertainment and sports events/venues				
	11200	112000	Travel related to attending/visiting cultural, entertainment and sports events/venues	n/a	n/a	n/a	n/a
1190			Attending/visiting sports, entertainment and cultural events/venues n.e.c.				

ICATUS 2005 activity				Category	MET	Wakeful-ness	Sitting/ lying
Code			Title				
	11900	119000	Attending/visiting sports, entertainment and cultural events/venues n.e.c.	LPA	2.40	yes	no
1211			**Visual, literary and performing arts (as hobby) and related courses**				
	12111	121110	Visual arts	LPA	2.75	yes	yes
	12112	121120	Literary arts	SB	1.30	yes	yes
	12113	121130	Performing arts (dance, music, theatre)	MVPA	4.00	yes	no
	1211x		Visual, literary and performing arts n.f.d.	LPA	2.75	yes	no
1212			**Technical hobbies and related courses**				
	12120	121200	Technical hobbies and related courses	LPA	2.80	yes	no
1213			**Playing games and other pastimes and related courses**				
	12131	121310	Solo games	LPA	2.90	yes	no
	12132	121320	Card games, board games	SB	1.50	yes	yes
	12133	121330	Computer games (including arcade and video games)	MVPA	3.05	yes	yes
	12134	121340	Social/group games	MVPA	5.00	yes	no
	12135	121350	Gambling	LPA	2.50	yes	yes
	1213x		Playing games and other pastimes n.f.d.	LPA	2.90	yes	yes
1220			**Travel related to hobbies, games and other pastimes**				
	12200	122000	Travel related to hobbies, games and other pastimes	n/a	n/a	n/a	n/a
1290			**Hobbies, games and other pastimes n.e.c.**				
	12900	129000	Hobbies, games and other pastimes n.e.c.	LPA	2.86	yes	yes
1311			**Participating in sports**				
	13111	131110	Walking and hiking; jogging and running	MVPA	5.05	yes	no
	13112	131120	Biking, skating, skateboarding	MVPA	7.00	yes	no
	13113	131130	Aerobics, yoga, weight-training and other fitness programmes	MVPA	3.50	yes	no
	13114	131140	Ball games, individual sports	MVPA	5.15	yes	no
	13115	131150	Ball games, team sports	MVPA	7.00	yes	no
	13116	131160	Water sports	MVPA	6.00	yes	no
	13117	131170	Winter/ice/snow sports	MVPA	7.00	yes	no
	13118	131180	Contact sports	MVPA	7.80	yes	no
1312			**Camping and other outdoor activities**				
	13121	131210	Camping	LPA	2.50	yes	no
	13122	131220	Horseback-riding	MVPA	5.80	yes	yes
	13123	131230	Pleasure drives; sightseeing	LPA	2.00	yes	yes
1320			**Travel related to indoor and outdoor sports participation and related courses**				
	13200	132000	Travel related to indoor and outdoor sports participation and related courses	n/a	n/a	n/a	n/a
1390			**Indoor and outdoor sports participation and related courses n.e.c.**				
	13900	139000	Indoor and outdoor sports participation and related courses n.e.c.	MVPA	5.80	yes	no
1411			**Reading**				
	14111	141110	Reading books	SB	1.30	yes	yes
	14112	141120	Reading periodicals	SB	1.30	yes	yes

ICATUS 2005 activity Code			Title	Category	MET	Wakeful-ness	Sitting/lying
	14119	141190	Reading other specified materials	SB	1.30	yes	yes
	1411x		Reading n.f.d.	SB	1.30	yes	yes
1412			Watching/listening to television and video				
	14121		Watching/listening to television	SB	1.15	yes	yes
		141211	Watching/listening to television (regular programming)	SB	1.15	yes	yes
		141212	Watching/listening to television (time-shifted programming)	SB	1.15	yes	yes
		141219	Other specified activities related to watching/listening to television	SB	1.15	yes	yes
		14121x	Watching/listening to television n.f.d.	SB	1.15	yes	yes
	14122		Watching/listening to video programmes	SB	1.15	yes	yes
		141221	Watching/listening to rented/purchased movies	SB	1.50	yes	yes
		141222	Watching/listening to rented/purchased video programmes other than movies	SB	1.15	yes	yes
		141229	Other specified activities related to watching/listening to video	SB	1.15	yes	yes
		14122x	Watching/listening to video programmes n.f.d.	SB	1.15	yes	yes
1413			Listening to radio and audio devices				
	14131	141310	Listening to radio programmes	SB	1.40	yes	yes
	14132	141320	Listening to other audio media	SB	1.40	yes	yes
	1413x		Listening to radio and audio devices n.f.d.	SB	1.40	yes	yes
1414			Using computer technology				
	14141	141410	Using computer technology for reading	SB	1.30	yes	yes
	14142	141420	Using computer technology for video/audio	SB	1.25	yes	yes
	14143	141430	Surfing the Internet; downloading, uploading	SB	1.30	yes	yes
	1414x		Using computer technology n.f.d.	SB	1.30	yes	yes
1420			Visiting library				
	14200	142000	Visiting library	LPA	1.80	yes	yes
1430			Travel related to mass media				
	14300	143000	Travel related to mass media	n/a	n/a	n/a	n/a
1490			Mass media n.e.c.				
	14900	149000	Mass media n.e.c.	SB	1.33	yes	yes
1511			Sleep and related activities				
	15111	151110	Night sleep/essential sleep	Sleep	0.95	no	yes
	15112	151120	Incidental sleep/naps	Sleep	0.95	no	yes
	15113	151130	Sleeplessness	SB	1.30	yes	yes
	1511x		Sleep and related activities n.f.d.	Sleep	0.95	no	yes
1512			Eating and drinking				
	15121		Eating meals/snack	SB	1.50	yes	yes
		151211	Eating a meal (including drinks taken with meal)	SB	1.50	yes	yes
		151212	Eating a snack (including drinks taken with snack)	SB	1.50	yes	yes
	15122	151220	Drinking other than with meal or snack	LPA	1.65	yes	yes
	1512x		Eating and drinking n.f.d.	LPA	1.58	yes	yes
1513			Personal hygiene and care				
	15131	151310	Personal hygiene and care	LPA	2.00	yes	no
	15132	151320	Health/medical care to oneself	SB	1.30	yes	yes

ICATUS 2005 activity<br>Code			Title	Category	MET	Wakeful-ness	Sitting/ lying
**1514**			**Receiving personal and health/medical care from others**				
	15141	151410	Receiving personal care from others	SB	1.30	yes	yes
	15142	151420	Receiving health/medical care from others	SB	1.30	yes	yes
**1515**			**Religious activities**				
	15151	151510	Private prayer, meditation, and other informal spiritual activities	SB	1.30	yes	yes
	15152	151520	Participating in religious activities (formal practice of religion)	LPA	2.00	yes	yes
**1516**			**Activities associated with resting, relaxing**				
	15161	151610	Doing nothing; resting, relaxing	SB	1.30	yes	yes
	15162	151620	Smoking	SB	1.30	yes	yes
	15163	151630	Reflecting/meditating, thinking, planning	SB	1.30	yes	yes
**1520**			**Travel related to personal care and maintenance activities**				
	15200	152000	Travel related to personal care and maintenance activities	n/a	n/a	n/a	n/a
**1590**			**Personal care and maintenance activities n.e.c.**				
	15900	159000	Personal care and maintenance activities n.e.c.	SB	1.30	yes	yes

Notes: MET: metabolic equivalent of task; n.f.d.: not further defined; n.e.c.: not elsewhere classified; SB: sedentary behaviour, LPA: light physical activity, MVPA: moderate-to-vigorous physical activity; n/a: not applicable
