# Supplementary material for: Standardised criteria for classifying the International Classification of Activities for Time-use Statistics (ICATUS) activity groups into sleep, sedentary behaviour, and physical activity
Source: Int J Behav Nutr Phys Act. 2019 Nov 14;16:106. doi: 10.1186/s12966-019-0875-5 (PMC6857154; doi:10.1186/s12966-019-0875-5)
Supplement: Supplementary file 3 — Additional file 3. 2016 ICATUS Assignment Table. Metabolic equivalent (MET) values, summary codes and movement categories assigned to 2016 Inte.rnational Classification of Activities for Time-Use Statistics (ICATUS) activities. [file 12966_2019_875_MOESM3_ESM.pdf]

Metabolic equivalent (MET) values, summary codes and movement categories assigned to International Classification of Activities for Time Use Statistics (ICATUS) 2016 activities

ICATUS 2016 activity Code		Title	Category	MET	Wakeful-ness	Sitting /lying
11		**Employment in corporations, government and non-profit institutions**				
	110	Employment in corporations, government and non-profit institutions	n/a	n/a	n/a	n/a
12		**Employment in household enterprises to produce goods**				
	121	Growing of crops for the market in household enterprises	MVPA	3.55	yes	no
	122	Raising animals for the market in household enterprises	MVPA	4.30	yes	no
	123	Forestry and logging for the market in household enterprises	MVPA	3.90	yes	no
	124	Fishing for the market in household enterprises	MVPA	4.15	yes	no
	125	Aquaculture for the market in household enterprises	MVPA	4.30	yes	no
	126	Mining and quarrying for the market in household enterprises	MVPA	5.50	yes	no
	127	Making and processing goods for the market in household enterprises	MVPA	3.00	yes	no
	128	Construction activities for the market in household enterprises	MVPA	4.50	yes	no
	129	Other activities related to employment in household enterprises to produce goods	LPA	2.03	yes	no
13		**Employment in households and household enterprises to provide services**				
	131	Vending and trading of goods in household enterprises	LPA	2.50	yes	no
	132	Providing paid repair, installation, maintenance and disposal in households and household enterprises	MVPA	3.00	yes	no
	133	Providing paid business and professional services in households and household enterprises	SB	1.50	yes	yes
	134	Transporting goods and passengers for pay or profit in households and household enterprises	LPA	2.50	yes	yes
	135	Providing paid personal care services in households and household enterprises	LPA	2.15	yes	no
	136	Providing paid domestic services	LPA	2.23	yes	no
	139	Other activities related to employment in households and household enterprises providing services	LPA	2.03	yes	no
14		**Ancillary activities and breaks related to employment**				
	141	Activities ancillary to employment	LPA	1.65	yes	yes
	142	Breaks during working time within employment	SB	1.30	yes	yes
15		**Training and studies in relation to employment**				
	150	Training and studies in relation to employment	LPA	2.40	yes	yes
16		**Seeking employment**				
	160	Seeking employment	SB	1.40	yes	yes
17		**Setting up a business**				
	170	Setting up a business	LPA	1.65	yes	yes
18		**Travelling and commuting for employment**				
	181	Employment-related travel	n/a	n/a	n/a	n/a
	182	Commuting	n/a	n/a	n/a	n/a
21		**Agriculture, forestry, fishing and mining for own final use**				
	211	Growing crops and kitchen gardening, for own final use	MVPA	3.55	yes	no
	212	Farming of animals and production of animal products, for own final use	MVPA	4.30	yes	no

Code	ICATUS 2016 activity Title	Category	MET	Wakeful-ness	Sitting /lying
213	Hunting, trapping and production of animal skins, for own final use	MVPA	3.30	yes	no
214	Forestry and logging, for own final use	MVPA	3.50	yes	no
215	Gathering wild products, for own final use	MVPA	3.50	yes	no
216	Fishing, for own final use	MVPA	4.15	yes	no
217	Aquaculture, for own final use	MVPA	4.30	yes	no
218	Mining and quarrying, for own final use	MVPA	5.50	yes	no
22	Making and processing goods for own final use				
221	Making, processing food products, beverages and tobacco for own final use	LPA	2.67	yes	no
222	Making, processing textiles, wearing apparel, leather and related products, for own final use	MVPA	3.00	yes	yes
223	Making, processing of wood and bark products, for own final use	MVPA	3.30	yes	no
224	Making, processing bricks, concrete slabs, hollow blocks, tiles for own final use	MVPA	4.75	yes	no
225	Making, processing herbal and medicinal preparations for own final use	SB	1.40	yes	yes
226	Making, processing metals and metal products for own final use	MVPA	4.50	yes	no
227	Making, processing of products using other materials for own final use	LPA	3.00	yes	no
229	Acquiring supplies and disposing of products and other activities related to making and processing goods for own final use	LPA	2.03	yes	no
23	Construction activities for own final use				
230	Construction activities for own final use	MVPA	4.30	yes	no
24	Supplying water and fuel for own household or for own final use				
241	Gathering firewood and other natural products used as fuel for own final use	MVPA	3.50	yes	no
242	Fetching water from natural and other sources for own final use	MVPA	4.30	yes	no
25	Travelling, moving, transporting or accompanying goods or persons related to own-use production of goods				
250	Travelling, moving, transporting or accompanying goods or persons related to own-use production of goods	n/a	n/a	n/a	n/a
31	Food and meals management and preparation				
311	Preparing meals/snacks	LPA	2.50	yes	no
312	Serving meals/snacks	LPA	2.50	yes	no
313	Cleaning up after food preparation/meals/snacks	LPA	2.50	yes	no
314	Storing, arranging, preserving food stocks	MVPA	3.00	yes	no
319	Other activities related to food and meals management and preparation	LPA	2.50	yes	no
32	Cleaning and maintaining of own dwelling and surroundings				
321	Indoor cleaning	MVPA	3.30	yes	no
322	Outdoor cleaning	MVPA	4.00	yes	no
323	Recycling and disposal of garbage	LPA	2.50	yes	no
324	Upkeep of in/outdoor plants, hedges, garden, grounds, landscape, etc.	MVPA	4.15	yes	no
325	Tending furnace, boiler, fireplace for heating and water supply	MVPA	3.40	yes	no
329	Other activities related to cleaning and upkeep of dwelling and surroundings	LPA	2.50	yes	no
33	Do-it-yourself decoration, maintenance and repair				
331	Do-it-yourself improvement, maintenance and repair of own dwelling	MVPA	3.30	yes	no
332	Installation, servicing and repair of personal and household goods including ICT equipment	MVPA	3.00	yes	no
333	Vehicle maintenance and repairs	LPA	2.65	yes	no
339	Other activities related to do-it-yourself decoration, maintenance and repair	MVPA	3.00	yes	no

Code	ICATUS 2016 activity Title	Category	MET	Wakeful-ness	Sitting /lying
34	Care and maintenance of textiles and footwear				
341	Hand/machine-washing	MVPA	3.00	yes	no
342	Drying; hanging out, bringing in wash	MVPA	3.00	yes	no
343	Ironing/pressing/folding	LPA	1.98	yes	no
344	Mending/repairing and care of clothes and shoes; cleaning and polishing shoes	LPA	2.40	yes	yes
349	Other activities related to care of textiles and footwear	LPA	2.40	yes	no
35	Household management for own final use				
351	Paying household bills	LPA	2.30	yes	yes
352	Budgeting, planning, organizing duties and activities in the household	SB	1.50	yes	yes
359	Other activities related to household management	LPA	2.30	yes	yes
36	Pet care				
361	Daily pet care	LPA	2.75	yes	no
362	Using veterinary care or other pet care services (grooming, stabling, holiday or day care)	LPA	2.30	yes	no
369	Other activities related to pet care	LPA	2.53	yes	no
37	Shopping for own household and family members				
371	Shopping for/purchasing of goods and related activities	LPA	2.00	yes	no
372	Shopping for/availing of services and related activity	LPA	1.65	yes	yes
38	Travelling, moving, transporting or accompanying goods or persons related to unpaid domestic services for household and family members				
380	Travelling, moving, transporting or accompanying goods or persons related to unpaid domestic services for household and family members	n/a	n/a	n/a	n/a
39	Other unpaid domestic services for household and family members				
390	Other unpaid domestic services for household and family members	n/a	n/a	yes	no
41	Childcare and instruction				
411	Caring for children including feeding, cleaning, physical care	LPA	2.08	yes	no
412	Providing medical care to children	LPA	2.00	yes	no
413	Instructing, teaching, training, helping children	LPA	2.20	yes	no
414	Talking with and reading to children	LPA	1.98	yes	yes
415	Playing and sports with children	MVPA	3.15	yes	no
416	Minding children (passive care)	SB	1.30	yes	yes
417	Meetings and arrangements with schools and child care service providers	SB	1.50	yes	yes
419	Other activities related to childcare and instruction	LPA	2.10	yes	no
42	Care for dependent adults				
421	Assisting dependent adults with tasks of daily living	MVPA	3.00	yes	no
422	Assisting dependent adults with medical care	MVPA	3.00	yes	no
423	Assisting dependent adults with forms, administration, accounts	SB	1.30	yes	yes
424	Affective/emotional support for dependent adults	SB	1.50	yes	yes
425	Passive care of dependent adult	SB	1.30	yes	yes
426	Meetings and arrangements with adult care service providers	SB	1.50	yes	yes
429	Other activities related to care for dependent adults	MVPA	3.00	yes	no
43	Help to non-dependent adult household and family members				
431	Feeding, cleaning, physical care for non-dependent adult household and family members including for temporary illness	MVPA	3.00	yes	no

Code	ICATUS 2016 activity Title	Category	MET	Wakeful-ness	Sitting /lying
432	Affective/emotional support for non-dependent adult household and family members	SB	1.50	yes	yes
439	Other activities related to care for non-dependent adult household and family members	MVPA	3.00	yes	no
**44**	**Travelling and accompanying goods or persons related to unpaid caregiving services for household and family members**				
441	Travelling related to care-giving services for household and family members	n/a	n/a	n/a	n/a
442	Accompanying own children	LPA	1.80	yes	no
443	Accompanying dependent adults	LPA	2.40	yes	no
444	Accompanying non-dependent adult household and family members	LPA	2.40	yes	no
**49**	**Other activities related to unpaid caregiving services for household and family members**				
490	Other activities related to unpaid caregiving services for household and family members	LPA	2.00	yes	no
**51**	**Unpaid direct volunteering for other households**				
511	Unpaid volunteer household maintenance, management, construction, renovation and repair	MVPA	3.35	yes	no
512	Unpaid volunteer shopping/purchasing goods and services	LPA	2.05	yes	no
513	Unpaid volunteer childcare and instruction	LPA	2.04	yes	no
514	Unpaid volunteer care for adults	SB	1.50	yes	yes
515	Unpaid volunteer unpaid help in enterprises owned by other households	LPA	2.30	yes	no
519	Other activities related to direct unpaid volunteering for other households	LPA	2.50	yes	no
**52**	**Unpaid community- and organization-based volunteering**				
521	Unpaid volunteer work on road/building repair, clearing and preparing land, cleaning (streets, markets, etc.), and construction	MVPA	4.75	yes	no
522	Unpaid volunteer preparing/serving meals, cleaning up	LPA	2.50	yes	no
523	Unpaid volunteer cultural activities, recreation and sports activities	MVPA	3.00	yes	no
524	Unpaid volunteer office/administrative work	LPA	2.30	yes	yes
529	Other activities related to community- and organization-based unpaid volunteering	LPA	2.50	yes	no
**53**	**Unpaid trainee work and related activities**				
530	Unpaid trainee work and related activities	n/a	n/a	n/a	n/a
**54**	**Travelling time related to unpaid volunteer, trainee and other unpaid work**				
540	Travelling time related to unpaid volunteer, trainee and other unpaid work	n/a	n/a	n/a	n/a
**59**	**Other unpaid work activities**				
590	Other unpaid work activities	LPA	2.45	yes	no
**61**	**Formal education**				
611	School/university attendance	SB	1.30	yes	yes
612	Extra-curricular activities	MVPA	3.00	yes	no
613	Breaks at place of formal education	LPA	1.65	yes	no
614	Self-study for distance education course work (video, audio, online)	SB	1.30	yes	yes
619	Other activities related to formal education	LPA	2.15	yes	yes
**62**	**Homework, being tutored, course review, research and activities related to formal education**				
620	Homework, being tutored, course review, research and activities related to formal education	SB	1.40	yes	yes

Code		ICATUS 2016 activity Title	Category	MET	Wakeful-ness	Sitting /lying
63		**Additional study, non-formal education and courses**				
	630	Additional study, non-formal education and courses	SB	1.40	yes	yes
64		**Travelling time related to learning**				
	640	Travelling time related to learning	n/a	n/a	n/a	n/a
69		**Other activities related to learning**				
	690	Other activities related to learning	LPA	2.00	yes	no
71		**Socializing and communication**				
	711	Talking, conversing, chatting	SB	1.50	yes	yes
	712	Socializing/getting together/gathering activities	LPA	1.90	yes	yes
	713	Reading and writing mail (including email)	SB	1.30	yes	yes
	719	Other activities related to socializing and communication	LPA	1.80	yes	no
72		**Participating in community cultural/social events**				
	721	Participating in community celebrations of cultural/historic events	LPA	1.80	yes	no
	722	Participating in community rites/events (non-religious) of weddings, funerals, births and similar rites-of-passage	LPA	1.80	yes	no
	723	Participating in community social functions (music, dance, etc.)	LPA	2.00	yes	no
	729	Other activities related to community participation	LPA	1.80	yes	no
73		**Involvement in civic and related responsibilities**				
	730	Involvement in civic and related responsibilities	LPA	2.30	yes	no
74		**Religious practices**				
	741	Private prayer, meditation and other spiritual activities	SB	1.30	yes	yes
	742	Participating in collective religious practice	LPA	2.00	yes	yes
	749	Other activities related to religious practice	LPA	1.65	yes	yes
75		**Travelling time related to socializing and communication, community participation and religious practice**				
	750	Travelling time related to socializing and communication, community participation and religious practice	n/a	n/a	n/a	n/a
79		**Other activities related to socializing and communication, community participation and religious practice**				
	790	Other activities related to socializing and communication, community participation and religious practice	LPA	1.80	yes	yes
81		**Attending/visiting cultural, entertainment and sports events/venues**				
	811	Attendance at organized/mass cultural events, and shows	MVPA	3.30	yes	no
	812	Attendance at parks/gardens	MVPA	3.30	yes	no
	813	Attendance at sports events	LPA	2.40	yes	yes
	819	Other activities related to attendance at cultural, entertainment and sports events	LPA	1.80	yes	yes
82		**Cultural participation, hobbies, games and other pastime activities**				
	821	Visual, literary and performing arts (as hobby)	LPA	2.75	yes	no
	822	Hobbies	LPA	2.40	yes	no
	823	Playing games and other pastime activities	LPA	2.90	yes	yes
	829	Other activities related to cultural participation, hobbies, games	LPA	2.00	yes	no
83		**Sports participation and exercise and related activities**				
	831	Participating in sports	MVPA	6.50	yes	no
	832	Exercising	MVPA	6.50	yes	no
84		**Mass media use** *				

Code	ICATUS 2016 activity Title	Category	MET	Wakeful-ness	Sitting /lying
841	Reading for leisure	SB	1.30	yes	yes
842	Watching/listening to television and video	SB	1.15	yes	yes
843	Listening to radio and audio devices	SB	1.40	yes	yes
849	Other activities related to mass media use	SB	1.30	yes	yes
85	Activities associated with reflecting, resting, relaxing				
850	Activities associated with reflecting, resting, relaxing	SB	1.30	yes	yes
86	Travelling time related to culture, leisure, mass-media and sports practices				
	Travelling time related to culture, leisure, mass-media and sports practices	n/a	n/a	n/a	n/a
89	Other activities related to culture, leisure, mass-media and sports practices				
890	Other activities related to culture, leisure, mass-media and sports practices	LPA	1.80	yes	no
91	Sleep and related activities				
911	Night sleep/essential sleep	Sleep	0.95	no	yes
912	Incidental sleep/naps	Sleep	0.95	no	yes
913	Sleeplessness	SB	1.30	yes	yes
919	Other sleep and related activities	Sleep	0.95	no	yes
92	Eating and drinking				
921	Eating meals/snack	SB	1.50	yes	yes
922	Drinking other than with meal or snack	LPA	1.65	yes	yes
93	Personal hygiene and care				
931	Personal hygiene and care	LPA	2.00	yes	no
932	Health/medical care to oneself	SB	1.30	yes	yes
939	Other activities related to personal hygiene and care	LPA	1.65	yes	no
94	Receiving personal care and health/medical care from others				
941	Receiving personal care from others	SB	1.30	yes	yes
942	Receiving health/medical care from others	SB	1.30	yes	yes
949	Other activities related to receiving personal and health/medical care	SB	1.30	yes	yes
95	Travelling time related to self-care and maintenance activities				
950	Travelling time related to self-care and maintenance activities	n/a	n/a	n/a	n/a
99	Other self-care and maintenance activities				
990	Other self-care and maintenance activities	SB	1.30	yes	yes

Notes: MET: metabolic equivalent of task; n.f.d.: not further defined; n.e.c.: not elsewhere classified; SB: sedentary behaviour, LPA: light physical activity, MVPA: moderate-to-vigorous physical activity; n/a: not applicable; * the collection of the contextual variable "Using an ICT device" is recommended.
